# Development of Clinical Weekly-Dose Teriparatide Acetate Encapsulated Dissolving Microneedle Patch for Efficient Treatment of Osteoporosis

**DOI:** 10.3390/polym14194027

**Published:** 2022-09-26

**Authors:** Jeeho Sim, Geonwoo Kang, Huisuk Yang, Mingyu Jang, Youseong Kim, Hyeri Ahn, Minkyung Kim, Hyungil Jung

**Affiliations:** 1 Department of Biotechnology, Yonsei University, Seoul 03722, Korea; 2 JUVIC Inc., No. 208, Digital-ro 272, Guro-gu, Seoul 08389, Korea

**Keywords:** osteoporosis, dissolving microneedle, teriparatide acetate, trehalose, centrifugal lithography, pharmacokinetic profile

## Abstract

Teriparatide acetate (TA), which directly promotes bone formation, is subcutaneously injected to treat osteoporosis. In this study, TA with a once-weekly administration regimen was loaded on dissolving microneedles (DMNs) to effectively deliver it to the systemic circulation via the transdermal route. TA activity reduction during the drying process of various TA polymer solutions formulated with hyaluronic acid and trehalose was monitored and homogeneities were assessed. TA-DMN patches fabricated using centrifugal lithography in a two-layered structure with dried pure hyaluronic acid on the base layer and dried TA polymer solution on the top layer were evaluated for their physical properties. Rhodamine-B-loaded TA-DMNs were found to form perforations when inserted into porcine skin using a shooting device. In addition, 87.6% of TA was delivered to the porcine skin after a 5-min TA-DMN patch application. The relative bioavailability of TA via subcutaneous injection was 66.9% in rats treated with TA-DMN patches. The maximal TA concentration in rat plasma was proportional to the number of patches used. Therefore, the TA-DMN patch fabricated in this study may aid in the effective delivery of TA in a patient-friendly manner and enhance medical efficacy in osteoporosis treatment.

## 1. Introduction

Osteoporosis, commonly described as a porous bone disorder, is a skeletal disorder characterized by the systemic impairment of bone density and strength and by the micro-architectural degeneration of bone tissue [1,2,3]. Abnormal quantitative and qualitative skeletal conditions of patients with osteoporosis negatively affect the mechanical properties of bone tissue and eventually result in bone fractures [4]. The properties of bone tissue are significantly related to the equilibrium between bone-resorbing osteoclasts and bone-forming osteoblasts, which enables the maintenance of sufficient bone density [5,6]. Dysregulation of several systemic circulating biomolecules, such as parathyroid hormone, steroid hormones, calcitonin, and vitamin D, as well as regionally secreted proteins such as osteoprotegerin, sclerostin, macrophage colony-stimulating factor, and various growth factors alter the equilibrium of osteoclast and osteoblast activity [6,7,8,9]. Anomalies in the secretion and expression of these modulators increase in an age-dependent manner; the prevalence of osteoporosis assessed via meta-analysis was estimated to be 18.3% worldwide in 2020, with 21% prevalence in people aged over 50 in the USA [10,11]. In addition to being the most common bone disease, the social cost of osteoporosis treatment in the USA is estimated to be USD 17 billion, and is predicted to increase to 25.3 billion by 2025 [12].

Effective treatment is essential to mitigate the suffering and socioeconomic burden on patients with osteoporosis. To treat osteoporosis, antiresorptive drugs such as bisphosphonates, which inhibit bone resorption, are used to reduce the bone turnover rate [13,14,15,16,17]. Despite successful retardation of osteoporosis progression, bisphosphonates are associated with osteonecrosis of the jaw and can cause several long-term adverse effects, including atypical femoral fractures related to oversuppression of bone turnover [18,19]. Recently, the anabolic drug teriparatide acetate (TA), known as recombinant human parathyroid hormone 1–34, which directly promotes bone formation by generating new osteocytes, has been introduced as a novel therapeutic agent for osteoporosis [20,21,22,23]. Distinct from antiresorptive drug treatment, patients with osteonecrosis of the jaw showed successful clinical outcomes after weekly administration of TA. In addition, adverse outcomes of long-term bisphosphonate treatment such as bone fracture were healed after usage of TA [24,25], and better bone mass and mineral density improvements at the lumbar spine and femoral neck were achieved with TA treatment than with bisphosphonate treatment [26].

Currently, commercial teriparatide products are administered to patients via 20 µg daily (Forteo; Eli Lilly Japan Co., Ltd., Kobe, Japan) and a 56.5 µg weekly (Teribone; Asahi Kasei Pharma Co., Ltd., Tokyo, Japan) subcutaneous injection of TA as a liquid formulation using hypodermic needles, which requires skilled medical professionals [27,28,29]. However, a daily or weekly visit to the clinic is inefficient for patients, and training patients for self-injection incurs considerable expense and time [30]. In addition, hypodermic needles can cause trypanophobia, pain, and irritation during injection, and the disposal of biohazardous sharp needles after injection can be expensive [31]. Therefore, a patient-friendly TA delivery method is necessary to overcome the limitations of hypodermic injections for osteoporosis treatment.

Among the various patient-friendly approaches, including iontophoresis, electroporation, sonophoresis, jet injection, and microneedle arrays (MNAs), MNAs are most promising alternative biomedical delivery systems to hypodermic injection [32,33,34,35]. Protein drugs carried by MNA, including biopharmaceuticals and vaccines, have been successfully released into the dermal layers via perforations created by MNA application [30,36,37,38]. Several aspects of MNA, such as minimal invasiveness followed by a low risk of infection, the possibility of self-administration and reduced adverse effects compared to hypodermic injection facilitate the easy delivery of drugs in a patient-friendly manner [36,37]. Recently, several studies have granted advanced transdermal delivery function to MNA by composing MNA with various polymers or materials, such as clay [39,40]. Therefore, effective TA delivery via MNA is anticipated to deliver TA in a more patient-convenient method, resolving the problems associated with hypodermic needle TA delivery for osteoporosis therapy.

TA-coated MNA was first introduced by laminating a TA-containing formulation on solid microneedles made of titanium or polylactic acid [28,41,42]. However, the biocompatibility of materials in solid MNA has been shown to increase the risk of inflammation, infection, and irritation, and redness at the perforations after application and accidental breakage of the solid MNA has been reported [43]. Therefore, the integration of TA into dissolving microneedles (DMNs) which encapsulate the drug into a biodegradable polymer matrix has been investigated for the delivery of TA without associated safety issues [44,45]. Because DMNs dissolve entirely in the skin, the adverse effects caused by the breakage of microneedles can be offset. Naito et al. showed pharmacokinetic profiles compatible with the conventional daily dose (20 µg) method using TA-loaded DMNs (TA-DMNs) [24]. Kim et al. developed TA-DMNs and analyzed the optimal formulation for rapid release and diffusion. In these studies, however, the dose of teriparatide for the once-weekly regimen (56.5 µg) and the pharmacokinetic profile for the clinical therapeutic window were not satisfactory. Moreover, the inevitable loss of TA activity during the DMN fabrication process and the effect of the stabilizer on minimizing loss were not evaluated. For clinical application with improved patient compliance, compensating for this decreased TA activity via TA-DMNs and a weekly dose (56.5 µg) of active teriparatide is required.

In this study, trehalose, which is widely known to induce protein stabilization by forming bonds between trehalose and protein, thereby slowing down the protein dynamics, was used as a stabilizer [46]. We evaluated the loss of TA activity during the drying of a mixture of TA and polymer for the DMN fabrication process. The activity loss of TA can be reduced to 75.4 ± 8.3% by adding 10% trehalose, which has previously been used as a TA stabilizer under stressful conditions [47]. The physical properties of TA-DMNs fabricated by centrifugal lithography (CL) were analyzed, and the appropriate mechanical strength of TA-DMNs to penetrate the skin barrier was determined [48]. Even penetration of all TA-DMNs and successful TA delivery were verified in porcine skin after TA-DMN application using a shooting device. Pharmacokinetic study of TA-DMNs in rats showed a bioavailability (BA) of 66.9% relative to the subcutaneous injection of TA, with minor irritation. Considering this BA and compensating for the activity loss of TA, we successfully formulated a weekly dose of TA able to satisfy the subcutaneous administration level, proving the efficacy and safety of this approach. The TA-DMN patch developed in this study is expected to enhance the efficacy of osteoporosis treatment without any patient discomfort and safety issues.

## 2. Materials and Methods

### 2.1. Preparation of TA-Loaded Polymer Solutions

A polymer mix of hyaluronic acid (HA) (32 kDa, Bloomage Freda Biopharm Co. Ltd., Jinan, China) was dissolved into distilled water, then agitated using a paste mixer (KM TECH Co., Ltd., Incheon, Korea) for homogenization. After adding synthesized TA (Anygen, Gwangju, Korea) and trehalose (Sigma-Aldrich, St. Louis, MO, USA) to the HA solution, a second agitation was performed using a paste mixer for homogenization at low temperature.

### 2.2. Evaluation of TA Activity in CL-Mimic Conditions

TA activity was assessed via reverse-phase high-performance liquid chromatography (RP-HPLC) under CL-mimic conditions, in which TA undergoes a drying process from droplets of matric polymer with TA. TA polymer solutions were dispensed as droplets on a polyurethane film (SLA, Gumi, Korea) using a robotic dispenser (ML-5000X; Musashi Engineering, Inc., Tokyo, Japan) and dried at 4 °C under vacuum to mimic the conditions of the CL. Dried droplets were then dissolved in phosphate-buffered saline (PBS; Life Technologies, Carlsbad, CA, USA) for reconstitution and analyzed via RP-HPLC using a separation module (Waters e2695; Waters Corp., Milford, MA, USA) and a dual λ absorbance detector (Waters 2487′ Waters Corp., Milford, MA, USA). A C18 column (COSMOSIL; 5 μm, 150 × 4.6 mm) was used for HPLC. Mobile phase A was prepared as 10% acetonitrile (Sigma-Aldrich, St. Louis, MO, USA) and 90% 0.2 M sodium sulfate (Sigma-Aldrich, St. Louis, MO, USA) and phase B was prepared as 50% acetonitrile and 50% of 0.2 M sodium sulfate with a flow rate of 1.3 mL/min and detection wavelength of 254 λ.

### 2.3. Evaluation of the Homogeneity of the TA-Loaded Polymer Solution

The homogeneity of the TA-loaded polymer solution was verified by comparing the relative standard deviation (RSD) of the mass of the solution, which was repeatedly dispensed by a robotic dispenser. HA aqueous solutions and formulated TA-loaded solutions were dispensed on the inside wall of a 1.5-mL Eppendorf tube using a robotic dispenser for constant time and pressure. The masses of the dispensed solutions were weighed using a weighing machine (Discovery DV125CD, Ohaus, Parsippany, NJ, USA), and the RSD values of each dispensed solution were calculated.

### 2.4. Fabrication of TA-DMNs

Primarily, the PU films were treated under plasma conditions for 7 min (PDC-32G-2, Harrick Plasma, Ithaca, NY, USA) to increase the bonding strength between the HA solution and PU films. The HA solution was then dispensed onto the PU films to form the base layer and dried at room temperature for 2 h. The TA polymer solution prepared in Section 2.1. was dispensed on the base layer, followed by TA-DMNs shaped by CL. During CL, centrifugation was performed for 3 min at 1300× *g* while vacuum conditions and a temperature of 4 °C were maintained.

### 2.5. Physical Properties of TA-DMNs

Microscopic images of the fabricated TA-DMNs were acquired using a bright field microscope (M165FC; Leica Camera AG, Wetzlar, Germany) and a digital microscope camera (DFC450C; Leica Camera AG) to assess the morphological properties of TA-DMNs. Morphological features of TA-DMNs, including the height and diameter of the tip and base, were measured using LAS v. 4.12 software. The mechanical strength of the TA-DMNs was assessed by measuring the minimal physical force required to create fractures using a force analyzer (Z0.5TN; Zwick Roell Inc., Ulm, Germany). The metal probe in the force analyzer descended at a speed of 2.0 mm/min to a single TA-DMN placed on the stage to measure the axial force needed to create a fracture in the TA-DMN.

### 2.6. Insertion of TA-DMNs into Porcine Skin

To evaluate the skin insertion properties of TA-DMNs, rhodamine B (Rho-B; Sigma-Aldrich, St. Louis, MO, USA) was added to the TA-loaded solution at 0.1% (*w*/*v*) to construct needle tips with fluorescence. Porcine skins (Cronex, Hwaseong, Korea) stored in a fridge at −20℃ were thawed in PBS for 30 min at room temperature. Thawed porcine skins were wiped and dried to remove the moisture on the surface. Then, Rho-B-loaded TA-DMN patches were applied thoroughly to porcine skin using thumb force or the force exerted by a shooting device and left for 5 min. After that, the patches were peeled off and the needle tips left in the porcine skin were imaged using bright-field microscopy. To assess whether penetration through the skin barrier by TA-DMNs was successful, porcine skin was stained with trypan blue (Sigma-Aldrich, St. Louis, MO, USA) and imaged via bright-field microscopy.

### 2.7. Release Profile of TA

To assess the release profile of TA in TA-DMNs, TA-DMN patches were inserted into porcine skin using a shooting device and left for 5 or 30 min. Then, the patches were peeled off and imaged using bright-field microscopy for subjective evaluation of TA-DMN dissolution after insertion. The remaining morphology of the TA-DMNs was analyzed, and the volume was evaluated using Autodesk Inventor 2022 (https://www.autodesk.com/, accessed on 4 August 2022). The remaining dose of TA on the patches was quantified using RP-HPLC analysis after dissolving the remaining TA-DMNs in PBS, followed by quantification to calculate the TA delivery rate to the skin.

### 2.8. In Vivo Pharmacokinetic Analysis after TA Delivery

Six-week-old male Sprague Dawley rats (SD rats) were purchased from Young Bio Inc. (Seongnam, Korea) and allowed seven days for acclimation. Three groups of SD rats were used for the pharmacokinetic study. All the dorsal regions of the SD rats were shaved for application of the TA-DMN patches. Group 1 was administered a subcutaneous injection of a formulation containing 60.6 μg of TA into the central part of the back using a 27 G hypodermic needle (Becton Dickinson, Franklin Lakes, NJ, USA), and served as the positive control. Group 2 received one TA-DMN patch containing 61.4 ± 1.3 µg of TA. Group 3 received two TA-DMN patches containing 122.8 ± 2.6 µg of TA. A home-made shooting device was used to apply the TA-DMN patches on the backs of the SD rats, and a dressing (Tegaderm; 3M Health Care, St. Paul, MN, USA) was used to cover the patches. In addition, a self-adherent bandage wrap (Coban; 3M Health Care, St. Paul, MN, USA) was used to secure the patches.

At 0, 0.25, 0.5, 0.75, 1, 2, 3.5, 4, 5, 6, and 24 h after subcutaneous administration of TA in group 1, 100 μL of blood was taken into a Microvette (SARSTEDT; Nümbrecht, Germany) from the saphenous vein, which was exposed after a minor incision of the tail by a surgical blade. Blood samples were collected in the same manner at 0, 0.5, 1, 2, 3.5, 4, 5, 6, and 24 h after TA-DMN patch application in group 2, and at 0, 0.5, 1, 1.5, 3, 4, 5, 6, and 24 h after TA-DMN patch application in group 3. The collected blood was left at room temperature for 30 min and centrifuged using a microcentrifuge (Hanil, Dajeon, Korea) at 1000× *g* for 10 min to separate the plasma. All separated plasma samples were transferred to 1.5-mL Eppendorf tubes and stored at −70 °C. TA concentrations in rat plasma were determined via enzyme-linked immunosorbent assay (ELISA) using a TA pharmacokinetics ELISA kit (SB-07-043; Somru Bioscience, Charlottetown, PE, Canada) and a microplate reader (Perkin Elmer, Waltham, MA, USA). Based on the determined TA concentrations in rat plasma, the areas under the concentration curve (AUC) were computed using the trapezoid area calculation method, and BA was evaluated to analyze the pharmacokinetic profiles.

### 2.9. Evaluation of Adverse Effects on Skin

The local TA-DMN patch-applied regions in rats were observed to evaluate any skin irritation and sensitization after skin perforation by TA-DMN insertion into the skin. The patch-applied area was imaged 5 and 20 h after TA-DMN insertion and the degree of erythema, wheal formation, and swelling were assessed. Erythema formation was analyzed by evaluating the visible redness of the applied skin surface. Wheal formation and swelling were simultaneously evaluated based on the skin surface elevation in the applied area.

### 2.10. Statistical Analysis

Graph Pad Prism 8 software (https://www.graphpad.com/scientific-software/prism/, GraphPad Software, Inc., San Diego, CA, USA, accessed on 1 August 2022) was used for the statistical analysis of all experimental results. Statistical analysis was performed using Student’s *t*-test or one-way analysis of variance (ANOVA) followed by post hoc analysis using Student’s *t*-test. All values were expressed as the mean ± standard deviation.

## 3. Results

### 3.1. Optimization of TA Polymer Formulation

#### 3.1.1. Effect of Trehalose on the Prevention of TA Activity during the Drying Process

**Figure 1:**
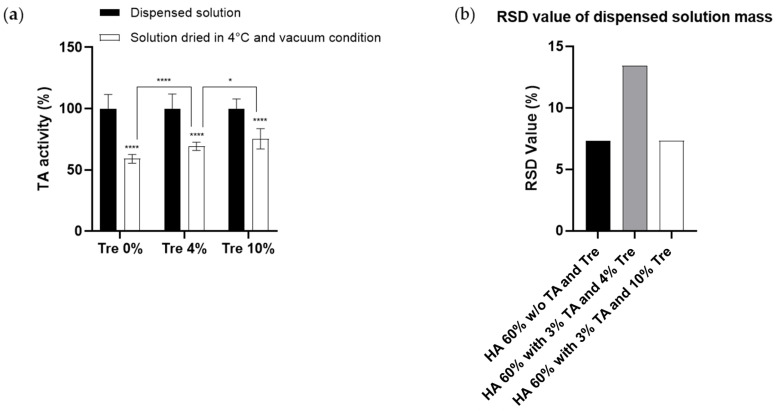
Teriparatide acetate (TA) polymer solution optimization based on TA activity maintenance during the drying process and solution homogeneity. (**a**) TA activity maintenance in TA polymer solution droplets dried at the CL-mimic conditions evaluated by HPLC analysis. Statistical significance was evaluated by ANOVA test and was set to *p* < 0.05, and values of * *p* < 0.05 and **** *p* < 0.0001 were considered to be statistically significant. n.s. means not significant. (**b**) Homogeneity of hyaluronic acid (HA) solutions without TA and trehalose and TA polymer solution.

Analyzing the reduction in TA activity during the fabrication process of TA-DMNs and the effect of trehalose on TA activity is essential for determining the dose of TA encapsulated in TA-DMNs. To analyze the effect of trehalose on TA activity during TA-DMN fabrication, 0, 4, and 10% trehalose concentrations containing TA polymer solutions were formulated and dried at low temperature in vacuum (CL-mimic) conditions. HPLC analysis was performed to assess the TA activity; as indicated in Figure 1a, TA activity significantly decreased to 59.0 ± 3.6% (*n* = 15) after drying without trehalose. Although the addition of trehalose did not completely prevent this activity loss, 10% trehalose reduced it to 75.4 ± 8.3% (*n* = 15).

#### 3.1.2. Homogeneity of TA Polymer Solution Droplets

Because TA-DMN was fabricated via CL, homogeneity of the TA polymer solution, which is a prerequisite of TA-DMN, is essential to dispense an even mass of TA polymer solution droplets on the PU patch, which leads to continuous morphology of every TA-DMN. The homogeneity of TA polymer solutions formulated with HA (60%, *w*/*v*) and TA (3%, *w*/*v*) and adjuvanted with 4 or 10% trehalose were compared to the homogeneity of HA (60%, *w*/*v*)-only solutions, which have sufficient homogeneity to fabricate DMNs with 8 × 8 arrays [48]. The RSD value of the masses dispensed from 60% HA solutions without TA and trehalose was 7.29%, while that of TA polymer solutions containing TA (3%, *w*/*v*) and adjuvanted with 4 and 10% trehalose with 60% HA was 13.45 and 7.32%, respectively (Figure 1b).

### 3.2. TA-DMN Fabrication by CL

**Figure 2:**
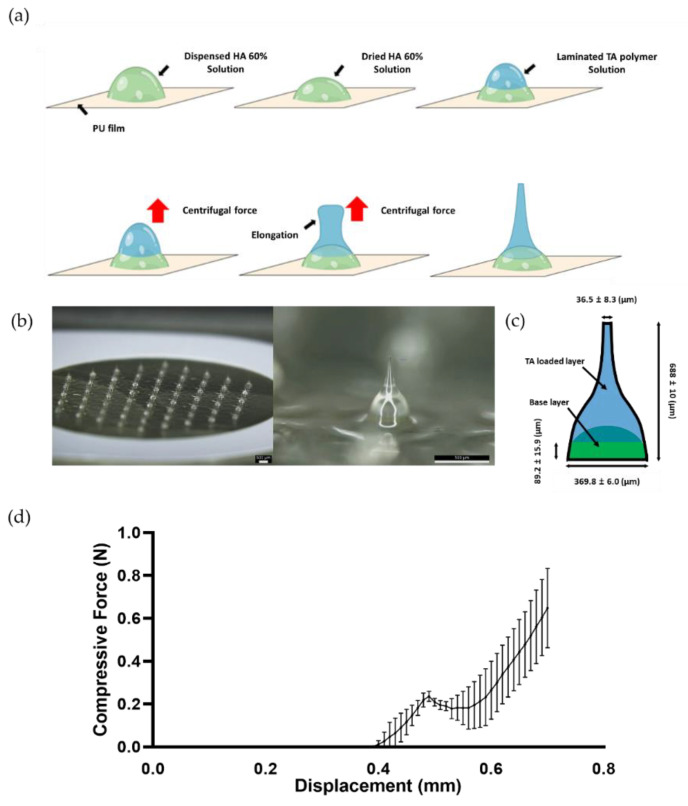
Schematic of the TA-loaded dissolving microneedle (TA-DMN) fabrication process and evaluation of physical properties of TA-DMNs. (**a**) TA containing solution droplet (blue) laminated on dried HA droplet (green) and centrifugal force exertion on two-layered droplets at low temperature and vacuum conditions. (**b**) Micrographic image of TA-DMN array on the patch and enlarged micrographic image of TA-DMN; the scale bar represents 500 µm. (**c**) Geometric specifications of TA-DMNs consisting of a TA-loaded layer (blue) and an empty base layer (green) (mean ± standard deviation, *n* = 3 in each type). (**d**) Fracture force analysis of TA-DMNs (*n* = 3).

As shown in Figure 2a, the TA polymer solution was laminated onto a dried droplet of HA solution, which is a prerequisite for the fabrication of TA-DMNs. The two-layered droplets were constantly rotated to generate upward centrifugal force and facilitate elongation. As the vacuum conditions for the drying process and centrifugal force exertion were maintained, the simultaneous thinning and solidification of the extended structure in TA-DMN formed the tip structures on the top of TA-DMNs. To deliver a fixed amount of TA into the skin, TA-DMNs were designed to have a two-layered structure incorporating a base layer without TA for accurate delivery [49]. Fabricated TA-DMNs were aligned with 61 hexagonal arrays on the PU patch (Figure 2b), and the total TA dose loaded on each patch, analyzed using HPLC, was 61.4 ± 1.3 µg, which was 0.86% of the mass of the 61 total TA-DMNs on the patch. Each TA-DMN showed a peak of 688 ± 10 µm, with negative curvature, a base diameter of 369.8 ± 6.0 µm, and a tip diameter of 36.5 ± 8.3 µm (Figure 2c). All top layers with TA were positioned over 89.2 ± 15.9 µm (Figure 2c). To evaluate whether the TA-DMNs had sufficient mechanical strength to penetrate the skin barrier, a compression test was conducted using a force analyzer. The fracture force of the TA-DMNs was found to be 0.237 ± 0.025 N (Figure 2d, *n* = 3).

### 3.3. Evaluation of In Vitro Skin Penetration of TA-DMNs

**Figure 3:**
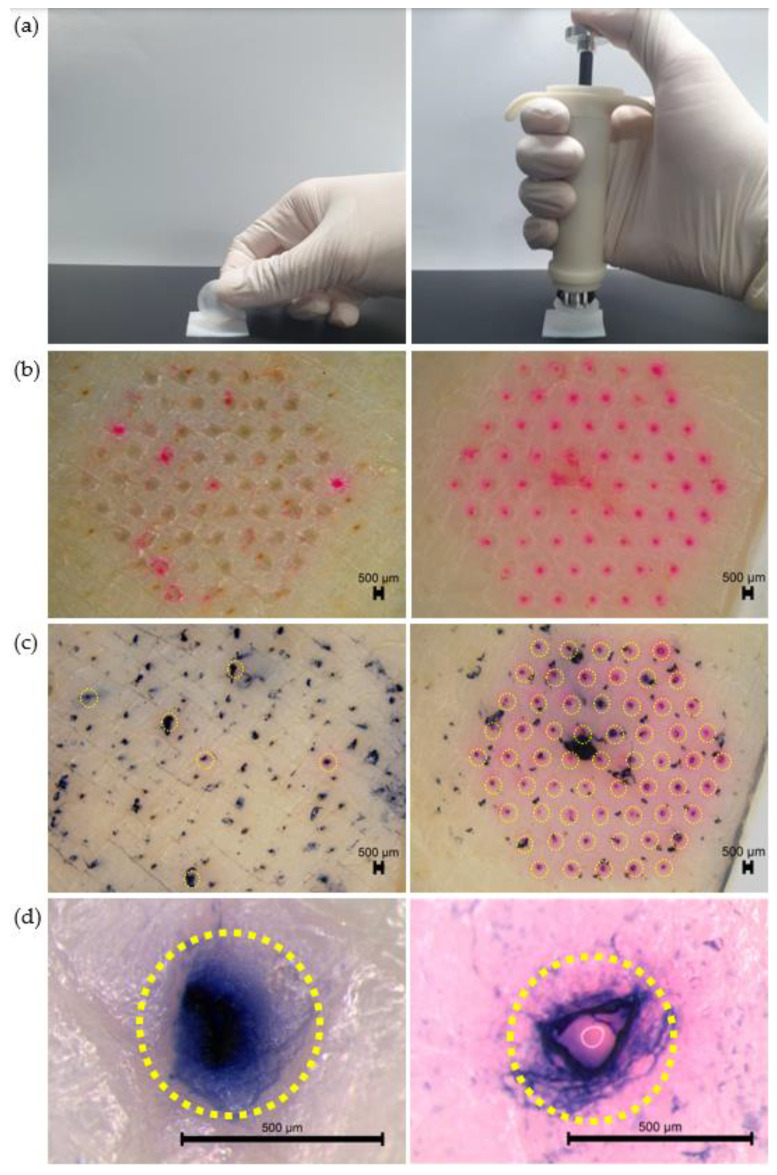
Analysis of the skin penetration of TA-DMNs applied using a shooting device or finger force: (**a**) TA-DMN patches application performed using finger force or a shooting device; (**b**) porcine skin inserted with Rhodamine B-loaded TA-DMNs using a finger force or a shooting device; (**c**) verification of porcine skin penetration by trypan blue staining; (**d**) enlarged images of perforated porcine skin. The black bar represents 500 µm.

A shooting device composed of a flat metal plate where a TA-DMN patch can be placed, a spring shooter to exert equal force, and a hand grip to hold (Figure 3a) was used to apply TA-DMNs on porcine skin. During application, the flat metal plate of the shooting device was placed 1 cm above the skin surface in parallel, then the patch was placed on the skin. TA-DMNs penetration of TA-DMNs into porcine skin applied using the shooting device and finger force with 0.1% of Rho B in the second layer was evaluated (Figure 3b). After 5 min of application of Rho B-loaded TA-DMNs on porcine skin using finger force, only six Rho B-loaded TA-DMNs were inserted. The other 55 points had a track indicating TA-DMN exertion, yet showed no Rho B dissolution or dispersion through the skin tissue. In contrast, after 5 min of application using the shooting device, 61 points of Rho B were observed, verifying that all 61 Rho B-loaded TA-DMNs were successfully inserted. Furthermore, Rho B-loaded TA-DMNs inserted into porcine skin were dyed with trypan blue solution to selectively visualize the TA-DMN penetrated area, thereby verifying whether the inserted TA-DMNs formed perforations on the skin surface (Figure 3c). All insertion points on the surface of the porcine skin subjected to finger force and the shooting device were amply dyed, and each dyed inserted point had a diameter of 106 ± 17 µm (Figure 3d).

### 3.4. In Vitro TA Release Profiles of TA-DMNs

**Figure 4:**
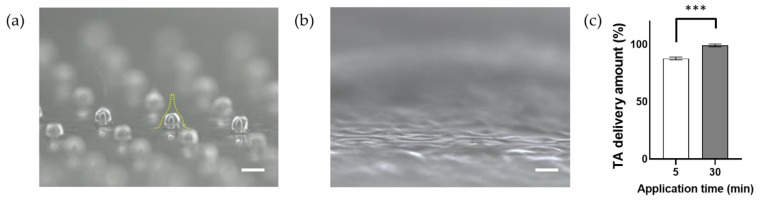
Transdermal delivery evaluation after TA-DMN patch application to porcine skin. (**a**,**b**) Images of remaining TA-DMNs after 5 and 30 min of application, respectively. Scale bar, 500 µm. (**c**) HPLC analysis of the amount of transdermal TA delivery after 5 and 30 min of application (*n* = 3, each). Statistical significance was evaluated by Student’s *t*-test, and values of *** *p* < 0.001 were considered to be statistically significant.

The morphologies of the remaining TA-DMNs were assessed, and the TA amount was evaluated via HPLC analysis after 5 and 30 min of application of TA-DMNs using a shooting device. The remaining volume of the TA-DMN patch was 60.6 ± 3.0% after 5 min of application (Figure 4a), and no TA-DMNs were left after 30 min of application of the TA-DMN patch on porcine skin (Figure 4b). The remaining of TA remaining on the patch was evaluated after 5 and 30 min of application via HPLC and found to be 12.4 ± 1.4 (*n* = 3) and 0.8 ± 1.2% (*n* = 3), respectively (Figure 3c). Therefore, over 99.2% of TA was delivered to the porcine skin when TA-DMNs were applied for 30 min.

### 3.5. Pharmacokinetic Profiles of TA-DMN Patch Applications

**Figure 5:**
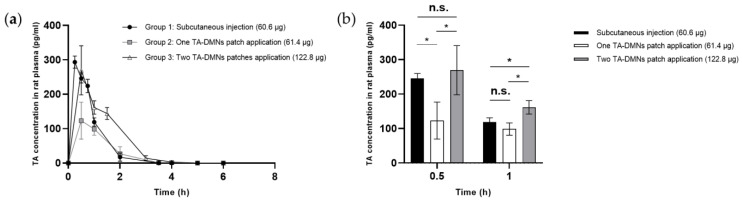
Pharmacokinetic profiles of TA in rat plasma after TA-DMN patch applications. (**a**) TA concentrations in the plasma samples of the rats in three experimental groups administered with TA in three different methods: (1) TA 60.6 μg dose subcutaneous injection (SC); (2) TA 61.4 μg dose TA-DMN patch application; and (3) TA 122.8 μg dose TA-DMN patch application. (**b**) Bar graph showing TA concentrations in the rat plasma collected 0.5 and 1 h after the administration of TA. Data represent the average ± standard deviation, and *n* = 3 for all groups. Statistical significance was set to * *p* < 0.05. n.s. means not significant.

To assess the systemic circulation of TA, SD rats were administered TA by three different measures: subcutaneous injection of liquid (Group 1, 60.6 µg TA administration), one TA-DMN patch (Group 2, 61.4 µg TA administration), and two TA-DMN patches (Group 3, 122.8 µg TA administration), all of which were applied using a shooting device. Pharmacokinetic profiles, including peak TA concentration (C_max_), time corresponding to the peak TA concentration, half-life of TA (t_½_), AUC, and BA, were evaluated after measuring the plasma TA concentration over time. As shown in Figure 5a, TA plasma concentration commonly surged after all three TA administration methods and then quickly decreased over 6 h, with a half-life of t_½_ ≈ 0.8 h. In the positive control (Group 1), SD rats subcutaneously injected with TA had a BA of 100%. As shown in Table 1, the BAs of groups 2 and 3 were 59.2 ± 18.1 (*n* = 3) and 66.9 ± 3.7% (*n* = 3), respectively, indicating no significant difference between the groups. However, when a doubled dose of TA was administered via TA-DMN patches in group 3, the AUC exceeded the AUC of group 1 (Figure 5a). TA concentrations in rat plasma at 0.5 and 1 h, which were the C_max_ and second highest TA concentration at specific time points, were compared (Figure 5b). Group 3 showed higher TA concentration than group 1 at 0.5 and 1 h.

### 3.6. Skin Adverse Reaction after TA-DMN Patch Application

**Figure 6:**
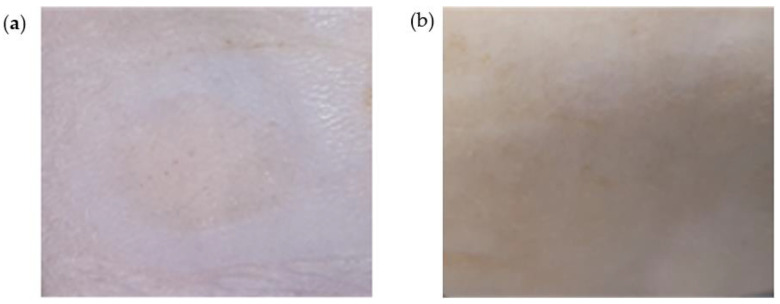
Images of skin stimulation and recovery after TA-DMN patch application: (**a**) image of the rat skin site 5 h after the application of the TA-DMN patch and (**b**) image of skin recovery 20 h after detachment of the TA-DMN patch.

TA-DMN application sites were observed in order to investigate any skin irritation and sensitization with respect to skin erythema, wheal formation, and swelling. Immediately after detachment and 20 h after detachment of TA-DMNs, erythema formation was assessed via visual redness assessment of the skin surface, and wheal formation with swelling was evaluated by verification of skin surface elevation. All rats treated with TA-DMN patches showed mild erythema without wheal formation and no swelling immediately after detachment of TA-DMN patches (Figure 6a). Twenty hours after detachment of the patches, all rats showed fully recovered skin with no redness (Figure 6b).

## 4. Discussion

In the present study, we evaluated the minimization of TA activity reduction during the drying process by adding trehalose to the formulation, which is a prerequisite for TA-DMNs. The TA-DMN fabrication process of viscous TA polymer solutions via shaping and drying induces a stressful environment that reduce TA activity. The addition of trehalose as a stabilizer significantly prevented TA activity reduction, which was increased with the increase in the concentration of trehalose. Because TA activity decreased to 75.4 ± 8.3% in the TA formulation with 10% trehalose after the drying process, loading an initial TA dose of approximately 80 μg before TA-DMN fabrication fulfilled the encapsulation of the weekly dose of TA in the TA-DMN patch. After assessing the reduction in TA activity, the homogeneity of TA polymer solutions was evaluated by comparing the RSD values of the dispensed polymer mass, representing homogeneity, to pure HA solutions, which have been proven to fabricate DMNs with constant morphology [48]. Because the TA-DMNs were fabricated via CL, they were shaped into microneedles from the dispensed droplets, with homogeneity of all the droplets’ masses being required to fabricate TA-DMNs with a regular morphology for uniform penetration and loading of uniform TA doses into the TA-DMNs. Therefore, we measured the mass of the droplets dispensed by the same air pressure and at the same time from the robotic dispenser, and the relative standard deviation from the average masses, that is, the RSD values, were compared. A higher RSD value from 4% trehalose-added TA polymer solution implies that adding less trehalose may cause incomplete prevention of TA aggregation and non-homogeneity in the TA polymer solution. TA polymer solution containing 10% trehalose showed similar homogeneity to pure HA (60%) solution; therefore, equal dispensing of TA polymer solution can be used to achieve a homogenous morphology of TA-DMNs.

To guarantee standard medical efficacy in patients, the delivery of accurate doses of drugs is crucial. However, owing to DMN morphology, DMN is resisted and pulled backward by the skin’s elastic force and tissue rigidity. For this reason, complete insertion of the DMN and delivery of an accurate dose of the drug encapsulated in the DMN are challenging. Therefore, TA-DMNs were fabricated into a two-layered structure with dried pure HA solution on the base layer and dried TA polymer solution on the top layer in order to concentrate the TA content close to the tip, where complete insertion may occur. Although a previous study demonstrated that the mechanical strength of DMNs can be reduced by encapsulating and increasing the drug dose [36], the TA-DMNs fabricated here had sufficient rigidity to penetrate the skin barrier.

Exerting uniform force on every TA-DMN followed by successful insertion is essential for administrating a constant dose of TA at each application. However, the magnitude and direction of the force exerted on each TA-DMN vary according to the person and the surface of the finger. Unequal force exertion and penetration issues were improved using a shooting device. The results showed that poor penetration performance and unequal forces exerted on TA-DMNs when finger force was used for application were significantly improved when the shooting device was used for application. Equal perforations imply that nearly uniform force was exerted on each TA-DMN; therefore, equal delivery of TA from every TA-DMN to the skin could be achieved using the shooting device.

Because a previous investigation reported patients’ preference for a short wear time of DMN patches [50], 5 min application of TA-DMNs was tested. Although a considerable volume of TA-DMNs remained after 5 min of application, a TA delivery amount of 87.6 ± 1.3% was achieved, possibly due to the two-layered structure of TA-DMN, which enabled the dissolution of a large portion of the top layer by the interstitial fluid in the skin. However, it can be speculated that incomplete dissolution of TA-DMN is deficient, meaning that an application time of more than 5 min is required. A delivery rate of over 97.8% with 30 min of application time achieved the complete dissolution of TA-DMNs, which implies that more dissolution occurred over 30 min.

BA decrease in TA-DMN was speculated to be due to the alteration of the delivery route from the subcutaneous to the intradermal region. Although a similar dose of TA-DMN (Group 2) compared to subcutaneous injection (Group 1) showed 59.2 ± 18.1% BA, there was no significant difference in the BA in group 3 (66.9 ± 3.7%), in which double the amount of TA was loaded. This implies that the same delivery route showed a similar BA, and thus the AUC values varied proportionally to the TA dose loaded into the TA-DMNs. In other words, the preferred pharmacokinetic profiles obtained from subcutaneous injection of TA can be achieved by altering the dose of TA in TA-DMNs. In this study, we showed that a double dose of TA-DMN exceeded the AUC of subcutaneous injection (Figure 5a) and that TA concentrations in rat plasma increased at 0.5 and 1 h. Although we loaded excess TA (122.8 µg) in the DMNs compared to the theoretical loading of TA for a weekly dose (84.5 µg, 100/66.9 ∗ 56.5 µg), we successfully delivered a weekly dose of TA via DMN, satisfying the subcutaneous administration level and thereby proving the efficacy of weekly application of TA-DMN patches for osteoporosis treatment. However, this requires further investigation for safety verification. HA and trehalose, which are components of TA-DMN, are United States Food and Drug Administration-approved biocompatible materials with proven safety in animal skin [51,52]. Because the mild reaction is merely cosmetic in nature and is not expected to be a significant inhibitor of safety [53], the tolerability of TA-DMNs was verified. This study suggests aspects of TA-DMNs for safety and successful delivery into the systemic circulation; therefore, further pharmacodynamic studies on the medical efficacy of this approach should be performed by compensating for the loss of TA activity via DMNs. In addition, a thorough evaluation of the immunogenic response of skin tissue towards the TA, such as by analyzing resected skin tissue through immunostaining, must be further investigated in order to ensure safety.

## 5. Conclusions

In conclusion, we developed a TA-DMN patch with a weekly administration regimen of TA by minimizing TA activity reduction during the fabrication process and compensating for the loss of TA activity. The equal penetrability of porcine skin and the efficient transdermal delivery shown by TA-DMN application using a shooting device signifies the potential for effective delivery of TA. In addition, successful transdermal delivery was demonstrated in an SD rat model without any safety issues, showing pharmacokinetic profiles compatible with the conventional method. These findings suggest the potential of using TA-DMNs for osteoporosis treatment without any patient discomfort. However, further study of TA delivery synergized with recent advanced transdermal delivery methods such as applying tissue-interlocking Candlelit-DMNs and composing MNA with biocompatible material with strong mechanical properties is required to improve the pharmacokinetic profiles of TA-DMNs [39,40,54,55].

## Figures and Tables

**Table 1:** Pharmacokinetic profiles of teriparatide acetate (TA)-administered rats.

	Group		
	1	2	3
Administered dose [μg]	60.6	61.4 ± 1.3	122.8 ± 2.6
C_max_ [pg]	293.4 ± 17.6	149.9 ± 45.9	338.8 ± 18.6
T_max_ (h)	0.3	0.7	0.5
t_1/2_ (h)	0.8	0.9	0.7
AUC (min × pg × mL^−1^)	255.9 ± 26.8	149.9 ± 45.9	338.8 ± 18.6
BA (%)	100 ± 10.5	59.2 ± 18.1	66.9 ± 3.7

## Data Availability

Data sharing not applicable.
